# A detailed theory of thalamic and cortical microcircuits for predictive visual inference

**DOI:** 10.1126/sciadv.adr6698

**Published:** 2025-02-05

**Authors:** Dileep George, Miguel Lázaro-Gredilla, Wolfgang Lehrach, Antoine Dedieu, Guangyao Zhou, Joseph Marino

**Affiliations:** Google DeepMind, London, UK.

## Abstract

Understanding cortical microcircuitry requires theoretical models that can tease apart their computational logic from biological details. Although Bayesian inference serves as an abstract framework of cortical computation, precisely mapping concrete instantiations of computational models to biology under real-world tasks is necessary to produce falsifiable neural models. On the basis of a recent generative model, recursive cortical networks, that demonstrated excellent performance on vision benchmarks, we derive a theoretical cortical microcircuit by placing the requirements of the computational model within biological constraints. The derived model suggests precise algorithmic roles for the columnar and laminar feed-forward, feedback, and lateral connections, the thalamic pathway, blobs and interblobs, and the innate lineage-specific interlaminar connectivity within cortical columns. The model also explains several visual phenomena, including the subjective contour effect and neon-color spreading effect, with circuit-level precision. Our model and methodology provides a path forward in understanding cortical and thalamic computations.

## Teaser

A generative vision model offers a detailed theory for cortical columns, thalamus, and computations in cortical circuits.

## INTRODUCTION

Understanding the functional logic of cortical microcircuits is an unsolved problem in neuroscience (*1*). In pursuit of this holy grail, advanced imaging and recording techniques are deployed to collect vast amounts of data from the brain (*2*, *3*). Deriving functional cortical circuits from this data is a formidable task, but required to understand how the brain works. To make substantial progress, cortical models need to satisfy three criteria simultaneously (*4*): They need to be computationally principled in addressing what the brain does, they need to have algorithmic realizations that solve problems that the brain is able to solve, and they need to have biological implementations that are well grounded in neuroanatomy and physiology such that they explain cognitive and neuroscience observations.

**Fig. 1.**
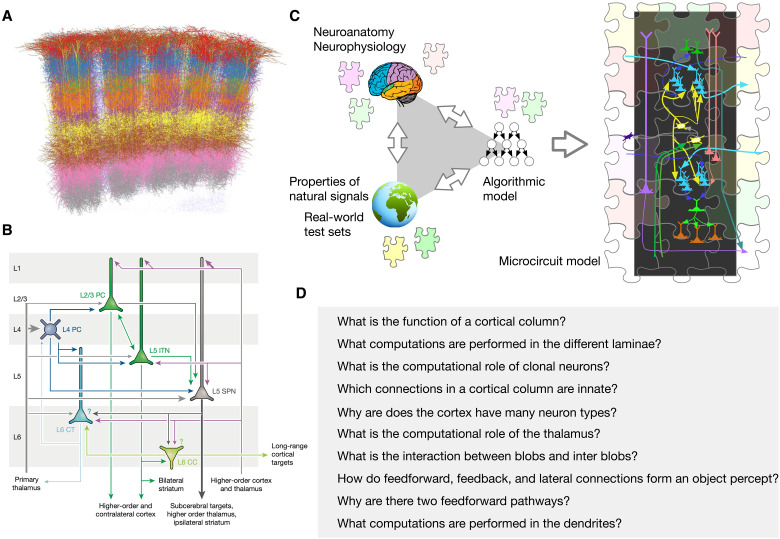
Different approaches to decipher cortical microcircuits. (**A**) A visualization of reconstructed cortical neurons shows the formidable complexity in understanding their functional logic. (Image credit: M. Oberlaender). (**B**) A diagram that summarizes cortical connections [reproduced with permission from (*58*)]. The summary is a useful sketch that does not correspond to a functional model. (**C**) Illustration of the triangulation methodology followed in this paper. The brain, the world, and computer science provide partial clues that can be used to construct an overall algorithmic model. The algorithmic model, along with neuroscience data, is then used to derive a functional circuit model. Just like the different pieces in a jigsaw puzzle constrain each other, the combination of algorithmic model and neuroscience data serve to constrain the space of microcircuit instantiations. (**D**) Examples of questions that can be asked and answered with a functional mathematical theory of cortical circuits. This paper answers those questions, and more.

Existing cortical theories and simulations fall short. Large-scale brain simulations (*5*) aspire to replicate biological details but lack computational and algorithmic grounding and struggle with solving real-world problems. While the theory of Bayesian inference (*6*, *7*) is gaining acceptance as an overarching framework to explain cortical computations, current incarnations primarily focus on describing “what” the brain does as opposed to “how” it does it (*8*). Another class of mechanistic models (*9*) has the advantage of biological realism but lacks an overall framework and lags in real-world tasks. Overcoming these shortcomings requires models that systematically investigate the algorithmic underpinnings of the wide array of questions experimental neuroscientists have answered regarding the connectivity, organization, and physiology of cortical circuits. Each of these experiments provides very important, but partial, clues regarding the functioning of the cortex. Coherent algorithmic models can exploit these cues for useful inductive biases, help fill in missing pieces, and resolve conflicting information (Fig. 1C).

Here, we derive a detailed and functional model of cortical microcircuits based on message-passing inference in recursive cortical networks (RCN), a generative model for vision (*10*). RCN is a neuroscience-based probabilistic graphical model (PGM) that systematically incorporated neuroscience findings as inductive biases to achieve state-of-the-art results on several vision benchmarks with greater data efficiency compared to prevalent deep neural networks. RCN is consistent with the overarching ideas of Bayesian inference, predictive coding, and free-energy minimization (*7*), but, in contrast to prior works that relied on simplistic models (*11*), the inference algorithms and representational choices of RCN are validated with real-world tasks (*10*). High-level Bayesian inference frameworks that do not confront the problem of tractability in realistic settings run the risk of being overly general and unfalsifiable (*8*), whereas testing on real-world settings enable the discovery of architectural and algorithmic details that matter. This distinction is crucial because the inductive biases and representational choices that enable efficient learning and inference also affect the wiring of cortical circuits.

Mapping RCN to a biological implementation results in a detailed model that delineates the role of innateness, learning, and dynamic inference computations in the connectivity and organization of cortical circuits. It assigns computational roles for connections in different laminae and columns, makes precise predictions about the interaction between interblobs and blobs, and assigns a functional role to the pathway through the thalamus. The model can also be used to understand the rationale for variations that arise from the same hodology (*12*). In addition, the model explains different visual phenomena like subjective contour percept (*13*, *14*), neon-color spreading (*15*), predicting neurophysiological activations and timing with circuit-level precision. Notably, all these observations are explained simultaneously as the natural by-product of doing “inference to best explanation” in a model that was learned for parsing a visual scene.

In computational and cognitive neuroscience, predictive processing of the sort we consider comes in two flavors: One can either assume that the brain uses continuous state-space generative models or “carves nature at its joints” in terms of discrete states. Committing to continuous or discrete latent variables has profound implications for the kind of message passing one would expect to see in cortical hierarchies. Most of the literature in this area assumes continuous latent variables, leading to schemes such as predictive coding that can be read in terms of Bayesian (e.g., extended Kalman Bucy or particle) filtering (*6*, *16*–*19*). However, when moving to discrete latent variables (*20*), the belief updating schemes have a different character and are generally formatted in terms of variational message passing and belief propagation (BP). Our focus will be on generative models with discrete latent variables, which may be more apt for higher-level perceptual inference, particularly in the visual and auditory domains. Complex problems such as perception, reasoning, and planning might require the more expressive inferences afforded by discrete state spaces—as opposed to the unimodal (Gaussian) posteriors often afforded by inference under continuous models, such as predictive coding. Having made this distinction, it should be noted that both predictive coding and BP can be formulated as message passing on factor graphs and, indeed, can be combined within the same generative model and ensuing inference scheme (*21*).

Our work is broadly compatible with and builds on the work in active inference (*22*, *23*). Several prior works exist in mapping BP to cortical anatomy (*11*, *24*–*27*), but they differ in the kinds of underlying computational models. For example, (*24*) considered tree-structured networks, with categorical variables in each node of the network. While this resulted in simplified inference, the network had limited expressivity in representing multiple overlapping objects or latent causes. Several other popular variants of predictive coding (*11*, *25*, *28*) also suffer from similar drawbacks of tree-structure networks or unimodal representations. In contrast, the RCN factor graph is heavily loopy, each latent cause being a binary random variable that densely interacts with the effects of other binary random variables. This results in distributed representations and the ability to represent simultaneous interacting latent causes. In addition, RCN also models rich lateral interactions between object parts and has factored contour and surface representations, details that are absent from previous attempts. These details were critical for the numerical performance of RCN in (*10*) and for the additional numerical simulations presented here.

This paper can be read as a review or synthesis that offers a particular process theory for BP in the brain based on a concrete computational model of visual inference (*10*), combining it with the neuroanatomy and neurophysiology of the visual system. This triangulation frames the functional architecture of the visual hierarchy and corticothalamic loops in terms of Bayesian belief updating under generative models with discrete state spaces. To illustrate the explanatory scope of the ensuing computational architecture, we will use numerical studies and simulations from the published literature (*10*), to which the reader is referred to for details.

## RESULTS

### Recursive cortical network

**Fig. 2.**
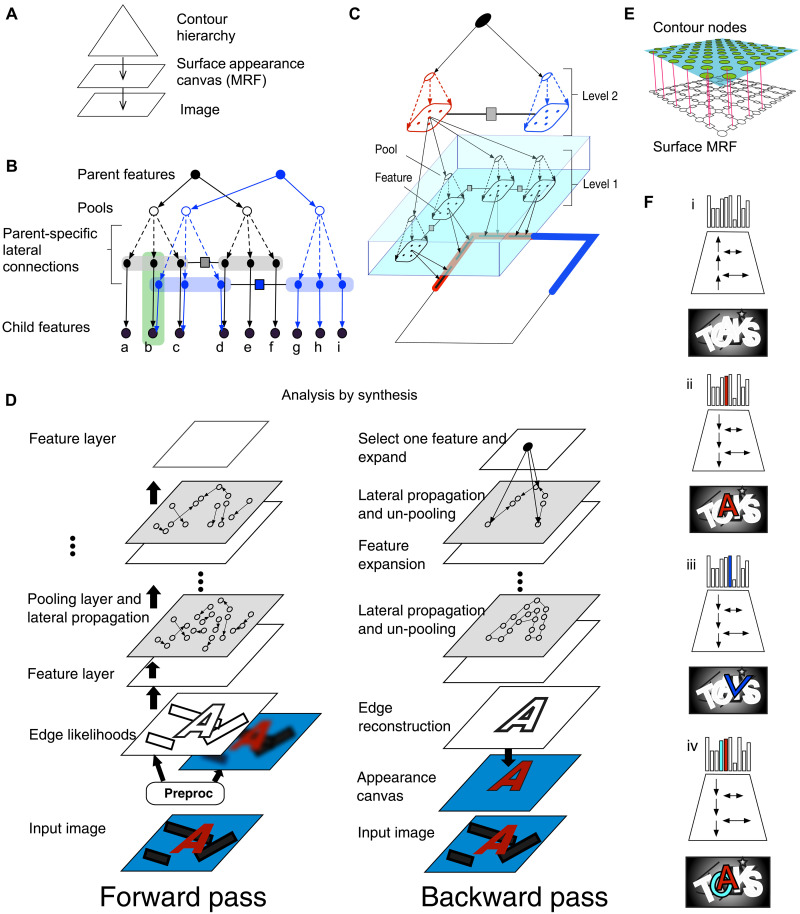
Structure of recursive cortical networks (RCN). (**A**) A compositional hierarchy generates the contours of an object, and a Markov random field (MRF) generates its surface appearance. (**B**) Contour hierarchy consists of features, pools, and laterals. Two subnetworks at the same level of the contour hierarchy keep separate lateral connections by making parent-specific copies of child features and connecting them with parent-specific laterals; nodes within the green rectangle are copies of the feature marked “b.” (**C**) A three-level RCN representing the contours of a square. Features at level 2 represent the four corners, and each corner is represented as a conjunction of four line-segment features. (**D**) Inference is achieved by passing messages along forward, backward, and lateral directions. (**E**) The surface appearance MRF. (**F**) Forward pass identifies object hypotheses in the scene, and backward and lateral pass segments it from the background for analysis by synthesis. Local hallucinations, e.g., the “v” in (iii), are explained away during parsing to obtain a global solution that best explains the evidence.

RCN is a structured PGM for vision consisting of a hierarchy of contour features that interacts with a surface appearance canvas to model images (Fig. 2A). The contour hierarchy contains alternating layers of feature variables and pooling variables. In Fig. 2B, each filled-in circular node is a binary random variable (feature), each empty circular node is a categorical random variable (pool), the elongated ellipses are sets of pooled features, and the squares between these sets are lateral connection factors that encode their compatibility. Pooling is an operation that provides invariance to local transformations, examples of which might include translations, scale variations, or rotations. In general, any variation in the input that occurs in temporal proximity could be pooled for creating an invariant representation.

The lateral connections between the pools—square “factor nodes” in Fig. 2 (B and C)—enforce contour consistency between the features in adjacent pools, thereby providing specificity. At the lowest level of RCN, the surface appearance canvas is implemented as a conditional Markov random field (MRF), as shown in Fig. 2E. The MRF limits conditional dependencies to its neighbors and encodes constraints regarding surface smoothness, i.e., surfaces are expected to vary smoothly when not interrupted by a contour (nodes in the upper layer) and discontinuously otherwise. Moving up the hierarchy, RCN decomposes an image into a hierarchy of increasingly invariant contours, resulting in a probabilistic model for the different objects in a scene. Figure 2C shows the hierarchical decomposition of a rectangle in terms of simple line segments at the bottom to more complex corner features at intermediate levels. Figure 2D is the graph corresponding to the representation of an “A” from a trained RCN. The graphs corresponding to higher level features share many of their lower level parts as shown in Fig. 2B (blue and black) so that a hierarchy of objects is constructed out of many shared parts. The learnable parameters in RCN are the contour features, the lateral connections that enforce contour continuity, and the surface-node parameters of the conditional MRF. These are learned using algorithms that approximate maximum likelihood learning as described in (*10*).

### Message-passing inference in RCN

RCN uses BP (*29*), a local message-passing algorithm for answering global inference queries in a graphical model. Although BP has no theoretical guarantees for producing the right solution in loopy graphs like RCN, appropriate scheduling and damping of messages have resulted in revolutionary empirical success (*30*).

Scene parsing in RCN is achieved through approximate maximum a posteriori (MAP) inference, also known as inference to best explanation, using the max-product (*29*) version of BP with a schedule inspired by biology (Fig. 2, D and F). The RCN model can be thought of as encoding the causal structure of the visual world through its hierarchy of latent variables, the nodes at the top of the hierarchy being objects that constitute scene, and the nodes at the intermediate layers being object parts. The goal of MAP inference is to assemble the combination of top-level and intermediate-level variables that best explain the evidence presented in the input. For a typical visual input that includes multiple objects on cluttered backgrounds and with mutual occlusion, assembling this best solution is an iterative process that involves forward, backward, and lateral message passing. Iteration is required because multiple latent causes can compete to explain the same evidence, and, sometimes, two mutually exclusive combinations can explain the evidence equally well. In RCN message passing, a fast-forward pass, which includes short-range lateral propagations, identifies nodes that are highly likely given the evidence. The backward pass focuses on highly active top-level nodes and includes longer-range lateral propagations. Analysis by synthesis (*31*) is naturally achieved through distributed message passing. The forward and backward passes assemble an approximate MAP solution that produces a complete segmentation of the input scene. See (*10*) for more details.

For people familiar with generative models expressed in terms of likelihoods and priors (e.g., partially observed Markov decision processes), the structure of the generative model in an RCN is determined by the existence of edges in the associated factor graph. This means that it is not necessary to parameterize probabilistic mappings between all latent states and outcomes because the requisite parameterization is implicit in the sparse connections and the nature of operations in each factor node.

### Neuronal implementation of message-passing inference

**Fig. 3.**
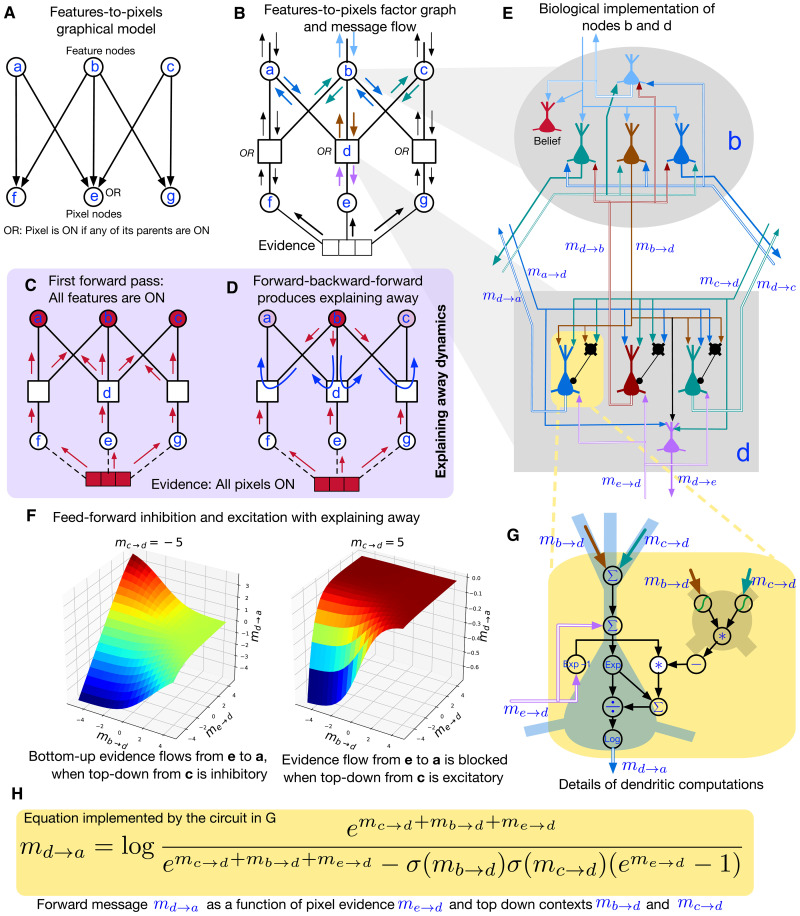
Neuronal mapping of message-passing in the “Noisy-OR” probabilistic graphical model (PGM), which is a part of recursive cortical networks (RCN) that represents how features and their components interact. (**A**) The Noisy-OR PGM with parent nodes representing features, and child nodes representing pixels. The multi-parent interactions at child nodes are modeled by the OR function. (**B** to **D**) Factor graph representation of the PGM in (A), with different message annotations. Corresponding to each edge, there are two belief propagation (BP) messages, one in each direction. Nodes consume the incoming messages to produce output messages. (C) First forward pass copies the bottom-up evidence to parent nodes. (D) Subsequent backward-forward passes produce explaining-away competition between parent features. (**E**) Neural implementation of message-passing in nodes *b* and *e*. (**F**) $m_{d\to a}$ as a function of $m_{e\to d}$ and $m_{b\to d}$ shows the excitatory-inhibitory interactions between the inputs. (**G**) Details of computation within an excitatory-inhibitory pair of neurons in *d*, to calculate $m_{d\to a}$ based on the BP equation in (**H**).

**Fig. 4.**
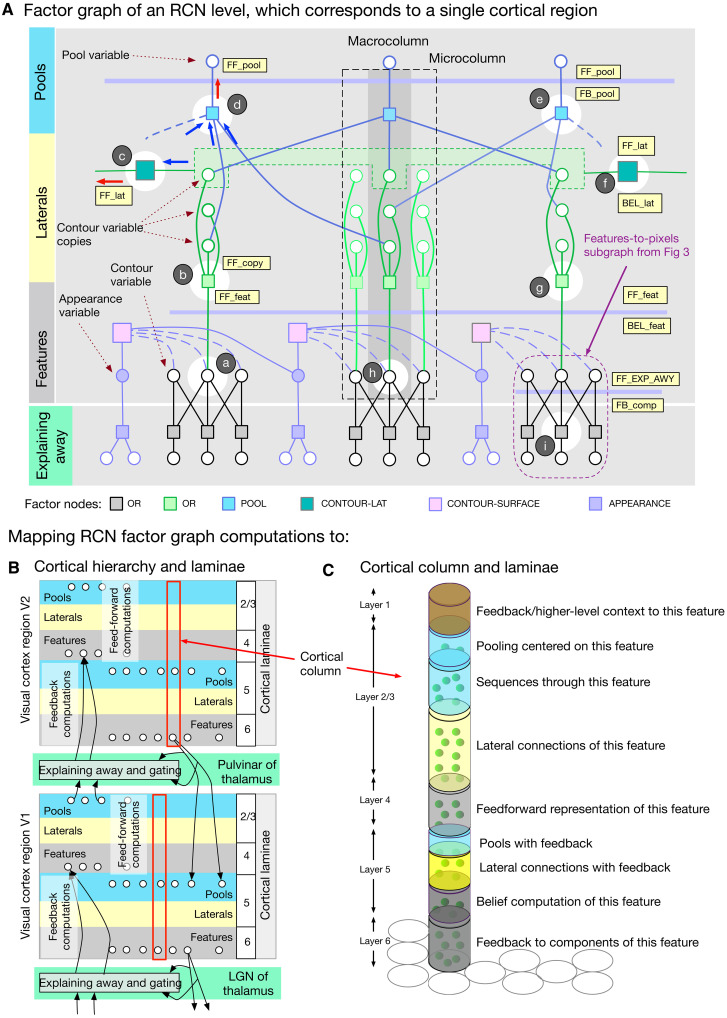
Stages of mapping recursive cortical networks (RCN) inference to cortical implementation. (**A**) Detailed factor graph of an RCN level. Thin horizontal lines indicate messages at different stages of inference, and these map to different laminae in a biological implementation. Variables and messages in a vertical slice as marked map to computations within a cortical column. (**B**) Conceptual cortical implementation of message passing. Messages in the different directions along the edge of a probabilistic graphical model (PGM) are implemented in biology using two different sets of neurons for features, laterals, and pools. Messages between different levels go through a stage that includes explaining away and gating. (**C**) Cortical column as a binary random variable that represents a feature or a concept, for example, an oriented line segment in V1 or the letter B, in IT. The different laminae in a column correspond to the inference computations that determine the participation of this feature in different contexts: laterally in the context of other features at the same level, hierarchically in the context of parent features, hierarchically as context for child features, and pooling/un-pooling for invariant representations.

To understand how RCN computations are mapped to biological implementation, we will first use a simple graphical model that is also a component of RCN. The Noisy-OR network model in Fig. 3A occurs at every level of the RCN hierarchy (Fig. 4) to represent object parts as features, and at the lowest level it maps features to pixels. The network in Fig. 3A has three feature nodes *a*, *b*, and *c*, and three pixel nodes *f*, *e*, and *g*. When a feature is ON, the pixels it is connected to are ON; feature *a* turns on pixels *f* and *e*; feature *b* turns on pixels *f*, *e*, and *g*; and feature *c* turns on pixels *e* and *g*. When a pixel node has multiple feature nodes connecting to it, they interact through a Noisy-OR mechanism that encodes the knowledge that the pixel is ON if any of its parent features are ON.

**Fig. 5.**
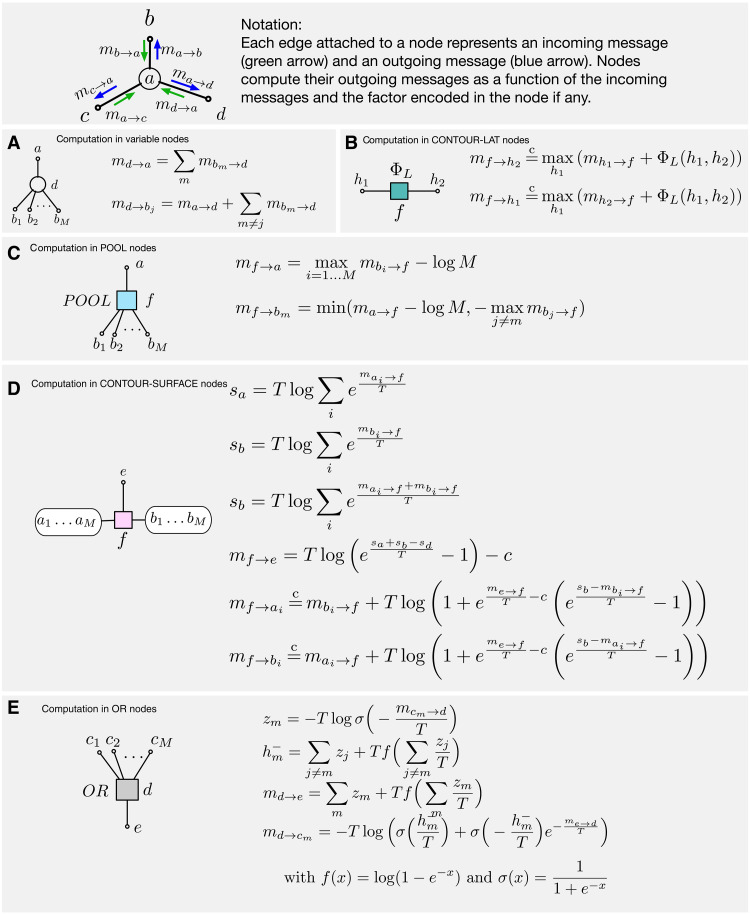
Belief propagation (BP) message computations in recursive cortical networks (RCN) factors and nodes. (**A** to **E**) All computations are in the log domain. Equations with a temperature *T* can do marginalization (*T* = 1), maximization (*T* = 0), or a soft maximization depending on the temperature setting. See Methods for derivations.

Figure 3B represents the same network as a factor graph where the random variable nodes, *a*, *b*, *c*, *f*, *e*, and *g*, are represented as circles and the interactions between these variable nodes are explicitly represented as square factor nodes; the factor node *d* represents that variable *e* is ON as Noisy-OR function of the variables *a*, *b*, and *c*. BP messages pass along the edges of the factor graph in both directions. Each node in the factor graph receives incoming messages from the edges connected to it and produces outgoing messages on those edges. The messages are labeled $m_{\text{source}\to \text{destination}}$. The message computations in the variable nodes are simple, as shown in Fig. 5A; the outgoing message on an edge is just the sum of incoming messages from every other edge. Computations in the factor nodes depend on the function implemented in the factor; computations for the Noisy-OR factor are shown in Fig. 5E.

Although this is a simple network, it captures many of the properties of parts-based representations in a hierarchy. Each feature variable can be thought of as representing part of an object as a latent cause, and the generative model specifies how the latent causes combine to create the visible pixels. The variable nodes in the factor graph are where probabilistic evidence can be presented, or they can be clamped ON or OFF to see their effects on other variables. Message passing is a parallel distributed mechanism by which the effect of perturbations in one variable node, whether it is evidence or clamping, propagates to other variables in the network. For example, consider setting the variable node *a* to ON and *b* and *c* to *OFF* and leaving the evidence at *f*, *e*, and *g* to be uncertain (equally likely to be ON or OFF). Message passing will then turn ON the pixel variable nodes *f* and *e* because the network specifies that turning *a* ON turns on *f* and *e*. One useful analogy is to think of message passing as the mechanism that keeps the network in equilibrium, propagating the effects of perturbations in the variable nodes to all the other nodes in the network. Unlike artificial neural networks where information propagates only in the feed-forward direction during testing, information propagates bidirectionally along all the edges in a factor graph. Inference queries in a factor-graph probe the states of some set of variable nodes in response to perturbations at the other set of variable nodes.

One inference query that is of interest is the MAP query that seeks the joint assignment of variable states that best explains the evidence. When evidence is presented, message passing–based inference in the network can settle on the MAP solution that selects the best assignment of ON/OFF states to all the nodes in the network. Consider the case when the evidence to the network is that of all pixels being ON as in Fig. 3C. Assume that, a priori, each of the features *a*, *b*, and *c*, is more likely to be OFF than ON. This means that, before the evidence is presented, the top-down messages to the OR factors reflect that the parent nodes are OFF. In the OR nodes, the feed-forward message to a parent is a function of the bottom-up message from the child and the top-down messages from all other parents. When all the parents are conveying that they are OFF, this function routes the bottom-up evidence to all the parent nodes, resulting in the first forward pass (Fig. 3C) broadcasting the evidence to each parent. Since the evidence offers independent support for each of the features *a*, *b*, and *c*, all the parent features will be ON after the first forward pass. Each parent then sends top-down messages to the OR factors connecting them to the child nodes reflecting the certainty that they are ON. The next forward message from the OR nodes will divvy up the bottom-up evidence such that parents that are more likely to be ON get more evidence, resulting in a competition between the parents for the bottom-up evidence. Since feature *b* being ON explains all the features being on, the backward pass and subsequent forward pass produce explaining away through the computations in the OR factors to diminish the evidence flowing to the nodes *a* and *c*, subsequently settling on the MAP solution that turns ON *b* and turns off *a* and *c*. Figure 3 (C and D) shows the propagation dynamics of explaining away between the feature nodes when all the pixels are ON. MAP assignments can also be bistable where two mutually exclusive combinations can explain the evidence approximately equally well. In this case, slightly perturbing the message propagation can cause it to find the other stable solution, for example, the combination of *a* = ON, *b* = OFF, and *c* = ON.

The top-down messages on to the OR nodes are context signals that affect how the feed-forward evidence is routed to the parents, and this contextual gating mechanism has interesting excitatory-inhibitory dynamics. The feed-forward message $m_{d\to a}$ from the OR factor *d* to the feature node *a* depends on the explaining-away computations in the OR factor *d* based on the top-down messages $m_{b\to d},m_{c\to d}$ it receives from the other parents, as shown in Fig. 3F. For example, when $m_{c\to d}$ is −5 as in the left panel, it is telling the OR node that parent *c* is OFF. In this case, how the evidence message $m_{e\to d}$ flows to parent *a* is affected by the top-down message $m_{b\to d}$. When $m_{b\to d}$ is −5, indicating that *b* is also OFF, $m_{d\to a}$ sends all the bottom-up evidence to *a*, showing a linear relationship. However, as the top-down message $m_{b\to d}$ becomes less negative, less of the bottom-up evidence flows to parent *a*. When $m_{c\to d}=5$ as in the right panel, most of the positive and negative evidence flow from *e* to *a* is blocked when parent *b* is also ON, and, when parent *b* is OFF, only negative evidence flows to parent *a*.

Figure 3 (E and G) shows details of how the message-passing equations for variable node *b* (Fig. 5A) and factor node *d* (Fig. 5E) are implemented using neurons. Since neurons are unidirectional, multiple neurons are required within each variable/factor node to convert the incoming messages to outgoing messages and to maintain the belief state of the node. In general, the message to each neighbor is implemented using a different neuron and carried by its axon. These neurons are shown using colors that correspond to the messages in Fig. 3B. Within a factor node, the synapses between incoming axons and the dendrites of outgoing axons and interneurons implement the computations required for the factor. In node *d*, the message to each parent is computed by a different neuron, and each of those neurons receives inputs from other parents. The blue, brown, and green neurons in node *d* in Fig. 3E calculate the outgoing messages to parents *a*, *b*, and *c*, respectively, using a circuit that involves inhibitory neurons and dendritic computations. The details of this circuit are shown in Fig. 3G, and this circuit implements the contextual gating transfer function in Fig. 3H. Implementing these equations require specific nonlinearities and dendritic computations (*32*) in these neurons.

Message passing can also be used to compute the marginal probability of each variable in the factor graph taking on a specific value, without committing an assignment to the other variables, given observed evidence. Whether message passing is computing marginalization (“sum-prop”) or MAP (“max-prop”) is determined by a specific change to the computations within the factors, which usually involves changing from a summation to a maximization. The equations in Fig. 5E for the OR factor have a temperature variable *T* that smoothly switches between sum-prop (*T* = 1) and max-prop (*T* = 0).

This simple network also serves to illustrate two different kinds of attention: spatial attention and top-down object-based attention. The Noisy-OR factors that are connected to every pixel in the factor graph have two parameters that affect how evidence is weighted; the “precision” parameter determines how likely the pixel (child) is to be ON when the feature (parent) is ON, and the “sensitivity” parameter determines how likely the pixel is ON because of noise when the feature is OFF. For example, if a portion of an image is expected to be noisy, evidence from that region can be downweighted by lowering the sensitivity of the pixels in that region. These parameters can be estimated during inference time for neighborhoods of pixels to have spatially varying gains for evidence, similar to the attention in predictive coding models (*25*, *33*). Another important form of attention that this network exhibits, but predictive coding models do not, is top-down object-based attention with explaining away. For example, setting the node *b* ON can be thought of as top-down attending to that feature. As we saw earlier, forcing node *b* to be ON will automatically diminish the flow of evidence to nodes *a* and *c* because the effects of these features overlap in the pixel space. Similarly, it is also possible to not attend to *b* by turning it off top-down, resulting in more evidence flow to alternative hypotheses that can explain the evidence.

### Anatomical mapping results

**Fig. 6.**
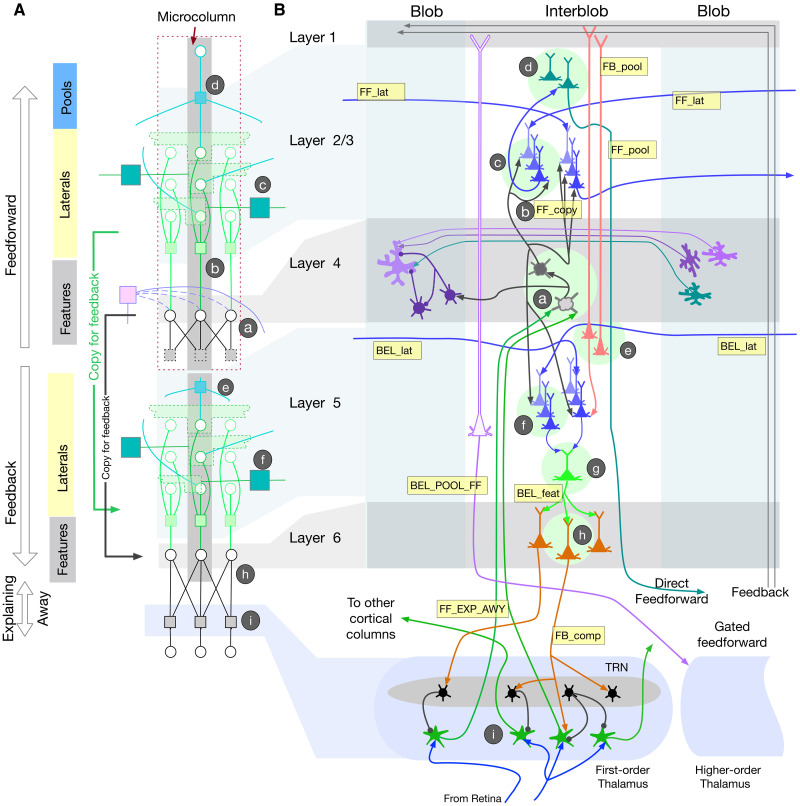
Biological implementation of recursive cortical networks (RCN) inference. (**A**) RCN factor graph segment for a microcolumn. Since Bio-RCN (**B**) requires different copies of neurons for forward and backward computations, the factor graph segment is replicated at the bottom left to show the correspondence between cortical layers and computations in the factor graph. The correspondence between RCN computations (**A**) and Bio-RCN (**B**) are annotated using circled letters a to i.

**Fig. 7.**
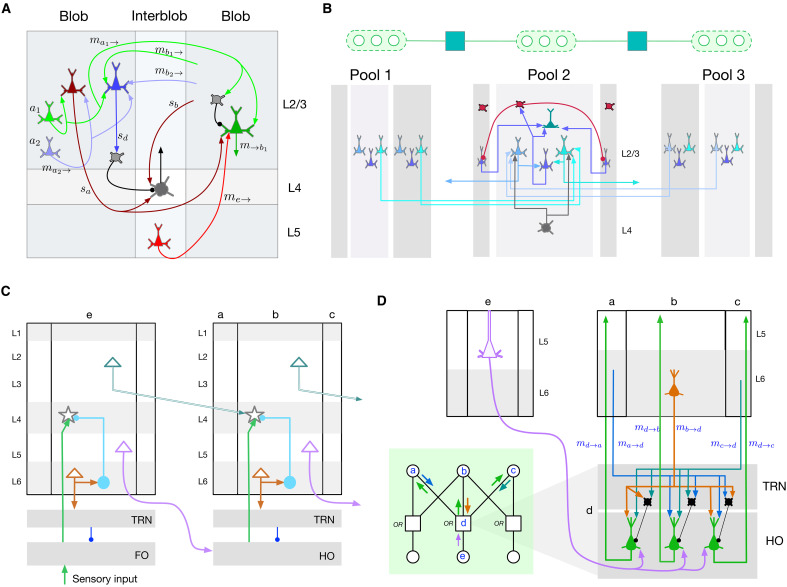
Cortical microcircuit motifs. (**A**) recursive cortical networks (RCN) equations predict that interactions between blob and interblob columns will include different forms of dendritic integration and gating. (**B**) Detailed microcircuits of contour-contour lateral connections and pooling. The dendrites of the neurons in layer 2/3 encode the compatibility factor (yellow node in the factor graph) between different pools. Columns in a pool also inhibit each other as part of lateral propagation. (**C**) Ineraction between cortical areas V1 and V2, gated through the thalamus. This pattern of connectivity is a repeating motif between different cortical areas. (**D**) In the green rectangle: factor graph showing the interaction between cortical columns *a*, *b*, and *c* in V2, with cortical column *e* in V1, through the factor *d*. Thalamic relay and thalamic reticular nucleus (TRN) microcircuit predicted by explaining-away computations in RCN and its connections to child and parent cortical columns. A feed-forward pathway originating in V1 layer 5 projects to the relay cells in higher order (HO) thalamus, which are gated by inhibitory TRN cells based on excitatory feedback projections from layer 6 in V2.

In the following sections, we describe how RCN computations (Fig. 5) map to cortical circuits, explaining or predicting their functional logic. We will refer to the cortical implementation by the name Bio-RCN and the abstract graphical model by the name RCN. We first describe the high-level mapping between RCN and Bio-RCN using Figs. 4 and 5 and then the details of the microcircuits. Details of the biological implementation are in Figs. 6 and 7.

### Overall organization of messages between cortical regions and the thalamus

Figure 4A shows details of one level of RCN in the form of a factor graph: Circles are random variable nodes, and rectangles are factors that encode the dependencies between variables. The variables in one RCN level are contour (black circles) or surface (filled circles) features, contour-copies (green circles) used in lateral connections, and pools (blue circles) that are locally invariant representations of contour segments. A node in the graph consumes incoming messages, processes them, and produces outgoing messages on all the edges it is connected to. The variable nodes and the factor nodes implement different computations on these messages, as described in Fig. 5. A pool variable of one level in RCN can participate in multiple higher-level features, and their competition is resolved during inference time using computations in the explaining-away block.

Figure 4B shows a coarse conceptual diagram of how message passing will be organized in a hierarchy when the feed-forward and feedback messages are implemented on separate pathways in cortical laminae. Within each level (cortical region), neurons in the feed-forward pathway process messages through a features-laterals-pools cascade to send messages to the next level, and the neurons in the feedback pathway do this in reverse, optionally combining feed-forward and feedback at different stages. Cortical laminae 2/3 and 4 typically process feed-forward information exclusively, while laminae 5 and 6 process feedback and produce “beliefs” that combine feed-forward and feedback. In Bio-RCN, these computations will be carried out in the dendrites and soma of networks of neurons. Neuronal outputs at different laminae correspond to messages computed at different stages in the RCN factor graph. Explaining away and gating are implemented in a different block that sits at the interface between two levels.

### Cortical micro-column as a binary random variable

A fundamental organizational unit of neocortex is that of cortical columns formed by synaptically connected vertical clusters of neurons (*34*), and the computational role of this columnar connectivity has remained a mystery. RCN assigns a functional and computational role to cortical columns: The whole column is viewed as a binary random variable, and the neurons in different laminae perform computations that infer the posterior state of this random variable from available evidence. The random variable represented by a cortical column can correspond to a “feature” or a “concept”—for example, an oriented line segment in V1 or the letter “B” in inferotemporal cortex (IT). The different laminae in a particular column correspond to the inference computations that determine the participation of this feature in different contexts: (i) laterally in the context of other features at the same level, (ii) hierarchically in the context of parent features, (iii) hierarchically as context for child features, and (iv) pooling/unpooling for invariant representations (Fig. 4C). During inference, their activities represent the contributions of this different contextual evidence in support of the feature being ON. The belief that the cortical column is ON itself is represented by specific neurons in specific laminae. In the factor-graph specification of RCN, the different aspects of a feature like lateral membership or pool membership are represented by binary variables themselves, but these binary variables are copies internal to the micro-column, and these copies represent the different contextual interactions of the feature-variable represented by the micro-column.

### Clonally related neurons in a cortical column

RCN assigns a functional rationale for clonally related excitatory neurons derived from a common progenitor. In the ontogenetic column, clonal neurons are known to share similar physiological functions, such as visual orientation selectivity (*35*–*37*). RCN uses copies of the same feature to represent its participation in different lateral and hierarchical contexts, and such a representation was shown to be parsimonious and advantageous in representing higher-order spatial or temporal relationships (*10*, *38*, *39*). The different copies in each group have identical bottom-up inputs or interlaminar inputs, while they differ in their lateral connections. In RCN, these vertical and top-down connections to the copies of a feature can be established a priori without any learning. In Bio-RCN, these a priori connections correspond to the vertical connections established in ontogenetic columns (e.g., axons marked FF_copy in Fig. 6) between clonally related neurons. Bio-RCN predicts that several interlaminar connections within a column can be established a priori as part of development without any learning and do not need to be modified further with experience, consistent with cortical measurements showing different levels of plasticity for different connections (*40*). Recent neuroanatomical observations about clonal neurons (*37*) are consistent with this proposal. Bio-RCN suggests that a cortical micro-column is initialized by hard-wired developmental programs to have integration of vertical inputs from different copies of the column’s feature and learned lateral connections from other cortical columns.

### Blobs and interblobs

Contour-surface factorization in RCN maps directly to interblob columns and cytochrome oxidase blobs in the primary visual cortex (*41*). The interactions between interblobs and blobs will be considered again in a subsequent section. Unless otherwise specified, descriptions of laminar processing within a column are with respect to interblob columns that represent contour features.

### Layer 4: Feed-forward feature evidence computation

According to Bio-RCN, layer 4 stellate cells compute the feed-forward evidence for features (FF_feat in Fig. 4A), obtained by the summation of bottom-up input messages FF_EXP_AWY along with the messages from contour-surface factors (Fig. 5A and annotation a in Fig. 4A). Computing this requires direct access to the bottom-up evidence, a requirement matched by spiny stellate cells in layer 4, which are the primary recipients of feed-forward inputs to a cortical region (*42*, *43*). The outputs from layer 4 are intracolumnar projections to layers 2/3 and 5, satisfying the requirements for subsequent computations in RCN.

This mapping assigns precise meaning to the thalamo-cortical axons and to the different stages of processing in layer 4. Each incoming message, carried by an axon from relay cells in the thalamus (green axons marked FF_EXP_AWY in Fig. 6B) is a scalar representing the log-likelihood ratio of a component of a feature being on. FF_feat is calculated in two stages, shown by gray neurons annotated a in Fig. 6, in Bio-RCN. The first stage computes the likelihood of contour features alone, ignoring the interaction with appearance blobs. The second stage combines this with inputs from the appearance blobs. A multistage computation within layer 4 with inputs from blob neurons is consistent with observations (*44*, *45*).

### Layer 2/3: Feed-forward lateral connections for contour continuity

Bio-RCN suggests that a subset of pyramidal cells in layer 2/3, annotated c in Fig. 6B, and their intercolumnar lateral projections implement contour continuity inference. Lateral factors in RCN (Fig. 4A, annotation c) encode a higher-order association field over contour features, and lateral message passing encourages contour-continuous solutions. In lateral propagation, the likelihood of each feature is calculated as a combination of bottom-up inputs from the features (FF_feat and FF_copy) and lateral messages (FF_lat) from other pools. In Fig. 4A, the green-colored nodes in the same column are “clones” that receive the same bottom-up input but have different lateral connections. The clones serve to encode long-range lateral dependencies (*46*, *47*), as opposed to just the first-order dependencies captured in pairwise models.

Layers 2 and 3 match the anatomical constraints for implementing these computations. Layer 2/3 pyramidal neurons receive feed-forward inputs from the layer 4 neurons (*12*), lateral inputs from co-circular pyramidal neurons in other columns (*48*, *49*), and send their axons across columns covering large distances, making patchy connections at their destinations (*42*, *50*–*53*). While the vertical interlaminar connections column can be specified a priori without learning, the recurrent lateral connections between pyramidal cells are learned from experience (*54*), consistent with the learning stages in RCN.

The blue neurons annotated c in layer 2/3 of Fig. 6 show the biological implementation. All of them receive the same bottom-up input FF_copy, axon branches of FF_feat calculated by layer 4 neurons. The two sets correspond to messages propagating in two different directions, and the copies within each set help to encode higher-order dependencies (*38*, *39*). Anatomical data support this proposal: Layer 4 spiny neurons make focused and dense connections to layer 2/3 pyramidal cells, with each spiny neuron innervating 300to 400 layer 3 pyramidal cells (*43*).

The lateral factor is a sparse matrix that encodes the compatibility between the features in the different pools. In Bio-RCN, this factor is realized in the dendritic trees of the neurons involved, implementing the computations in Fig. 5D. The lateral factors treat each pool as a categorical random variable that introduces mutual exclusivity between the constituent binary feature random variables (light green region in Fig. 4A). This operation involves mutual inhibition and normalization between the features that form constituents of the pool. We suggest that somatostatin-expressing inhibitory neurons in the superficial layers of the cortex are suited for this per-pool normalization, consistent with recent findings of (*55*). Figure 7B shows a more detailed implementation of the lateral connections between two pools, including their inhibitory fields.

### Layer 2/3: Pooling and feed-forward output

A subset of the complex pyramidal cells in layer 2/3 (*56*, *57*) matches the pooling operation in RCN. Pooling, a special case of which is pooling over translations of the same feature, gives invariance to local transformations. In general, pools are flexible and specified nonparameterically through the specific features they connect to. In the feed-forward pass, the POOL factor nodes (Fig. 4A, annotation d) implement a maximum operation over the input messages and produce the FF_pool output (Fig. 5C).

The green neurons annotated d in layer 2/3 or Fig. 6B compute the pooling output in Bio-RCN. Multiple clones of this neuron, all with the same bottom-up inputs, participate in different higher-level features. The pool neurons in a particular column represent the pools that are “centered” at that column. Pooling neurons need to receive trans-columnar inputs from features that are transversal, for example, different translation of a horizontal line. Similar to contour continuity laterals, connections for pooling need to integrate information from multiple columns. The pyramidal neurons in layer 2/3 that receive inputs laterally from other feature columns are ideally suited for performing these computations. The outputs of pooling neurons FF_pool is the direct feed-forward output of the region that is sent to the next level and will be considered in detail in a subsequent section.

### Layer 1: Feedback connections from higher levels

Feedback messages convey top-down certainty about the pools in lower-levels using a scalar corresponding to each pool (FB_pool messages in Fig. 4A). In Bio-RCN (Fig. 6), these feedback lines rise to layer 1 and extend horizontally (*58*–*60*). Pyramidal neurons in different columns and laminae can access these messages by extending their distal dendrites vertically into layer 1.

### Layer 2/3 or layer 5: Feedback-based unpooling

Unpooling is the reverse operation of pooling, where the evidence of a parent feature being ON is distributed to all its component pools at lower level (Fig. 4A, annotation e). Neurons in a cortical implementation (annotated e in layer 5 in Fig. 6B) will need to receive feedback information through their apical dendrites, send their outputs across columns to all the features that are part of the pool, and terminate in laminae that are involved in the next stage of feedback processing. A class of neurons in layer 3 and layer 5 both matches the requirements for this. The descending projections from layer 2/3 to layer 5 is a classical pathway that has been confirmed in multiple studies (*61*, *62*) and is known to control the gain of layer 5 outputs (*63*, *64*). Alternatively, the same computations could be implemented in a class of layer 5 pyramidal neurons with similar connectivity constraints (*43*).

### Layer 5: Lateral propagation with top-down inputs

Bio-RCN predicts that a set of neurons in layer 5 implements lateral propagation similar to the layer 2/3 pyramidal neurons. Feedback computations of contour-continuity laterals are similar to that of feed-forward lateral computations, except for the addition of top-down information. Top-down messages act as a priors on the pools at the lower level and determine which pools in the children are ON/OFF. The specific feature column that is to be turned ON within a pool is then determined as the one most compatible with its neighboring pools based on lateral message passing (annotation f in Figs. 4A and 6B).

Layer 5 pyramidal cells match the constraints for this because they have long-range lateral arborizations within layer 5 (*60*, *61*) and can receive feedback information through their apical dendrites in layer 1 or layer 3 (*43*, *65*). Layer 2/3 pyramidal neurons are a direct source of excitatory input to layer 5, and the cortico-cortical pyramidal neurons in layer 5 A/B are known to have lateral arborizations similar to that of layer 2/3 pyramidal cells, matching the requirements for these being a copy of the contour-continuity lateral connections (*12*). Although detailed data about layer 5 inhibition fields are missing, our mapping predicts that these layer 5 neurons will also have inhibition fields as those suggested for layer 2/3 neurons (*55*).

### Layer 5: Pool belief as a feed-forward output through the thalamus

RCN provides an explanation for the logic of parallel feed-forward pathway in addition to the direct cortico-cortical feed-forward pathway originating from layer 2/3 (*66*, *67*): The cortico-thalamo-cortical pathway provides mechanisms for explaining away, where thalamus implements the gray OR factor annotated i in Fig. 4A (equations in Fig. 5E). Explaining away is a form of gating mechanism where feed-forward messages are affected by feedback messages. The same neuronal apparatus that supports explaining away can also be used for other forms of gating that includes top-down object-based attention (hard explaining away), suppression, or graded gating. To be consistent with a direct feed-forward pathway that conveys evidence about pools, the thalamic feed-forward pathway needs to be about pools as well. The layer 5 pyramidal neurons projecting to the thalamus also receive feedback information from layer 1. Therefore, the feed-forward message through the thalamus is likely to be the belief in pools, which combines both feed-forward and feedback evidence.

The purple-outlined neuron in Fig. 6B shows the proposed cortical implementation. The pool likelihood neurons that project from layer 2/3 for a direct feed-forward connection (green neurons marked d in Fig. 6B) also synapses in layer 5. A subset of pyramidal neurons in layer 5 that receive feedback information through their dendrites in layer 1 can compute pool beliefs by combining input from this pool likelihood axon branch. According to Bio-RCN mapping, these layer 5 neurons project to the thalamus, where they take part in explaining away computations and gating, before projecting to the next level in the hierarchy. BP allows for sending either likelihoods or beliefs as feed-forward messages (*68*) because the next level in the hierarchy can derive likelihoods from the belief message by subtracting out the feedback message originating from that region. In Bio-RCN, these computations are implemented as part of the explaining away in the thalamus and will be considered in more detail in a subsequent section.

In the current version of RCN, a child region cannot turn on pools that are not turned on by the parent region, a constraint that we expect to be relaxed in future versions to support more dynamic assemblies based on lateral evidence. This would require projections from the lateral computations, shown as dotted axons from the blue pyramidal neurons in layer 5 of Fig. 6. The need to turn ON pools not supported by top-down lends further support to need for the existence of a pool belief neuron.

### Layer 5: Calculation of feature beliefs

Feature belief is the summary output of a column on its status (ON or OFF) incorporating all available evidence (BEL_feat in Fig. 4C). The final step in this computation is a summation of evidence from feature copies that participate in different lateral connections. A subset of neurons in layer 5, which are downstream from the lateral computation circuit (green neurons annotated g in layer 5 of Fig. 6) match the connectivity requirements for this computation (*63*). These neurons project to subcortical circuits (*12*), arguably because it is advantageous to drive actions from beliefs.

### Layer 6: Feedback message to child regions

Connections of layer 6 neurons (*62*) are consistent with the anatomical requirement for computations of RCN feedback messages from one level to its children (annotation h in Fig. 4A). Feedback messages for child regions are the reverse of the feed-forward computation where the belief of a feature is unpacked into its components and sent to the corresponding child regions. The three brown neurons annotated h in layer 6 of Fig. 6 correspond to the feedback messages to the three components of the feature represented by this column. Many feed-forward thalamo-cortical axons that synapse in layer 4 also send axon branches to layer 6 (*60*). The details of this layer 6 projection are unknown, but RCN can suggest one explanation where these projections help to subtract out the feed-forward influence from the feedback projections.

### Functional role of thalamus: Explaining away, gating, top-down attention, and binding

The functional logic of the thalamic pathway has been a source of enduring mystery in neuroscience (*69*). Anatomical data show two feed-forward circuits: a direct cortico-cortical connection from layer 2/3 and an indirect cortico-thalamo-cortical connection from layer 5 (*66*, *70*, *71*). The thalamus also receives feedback connections from layer 6 neurons in the higher level (*66*, *72*, *73*). The higher-level neurons that send feedback projections from layer 6 also project back to layer 4 of the same region via an inhibitory circuit as shown in Fig. 7C. Feedback projections from layer 6 project to the thalamic reticular nucleus (TRN) of the thalamus and make precise connections that modulate the feed-forward pathway.

RCN message passing offers an integrated explanation for functional role of the thalamus, including many of the details of the thalamic circuits, and connections spanning multiple cortical regions. The gray-colored OR factors in RCN (Fig. 2C) encode how multiple features interact while generating the same lower-level components. Such multicause interactions produce explaining-away effects during inference where the strength of one hypothesis is diminished when the same cause can be explained by the presence of another. These OR factors are also the interfaces between sensory input and the first level and between different levels. According to RCN, the thalamus implements this OR factor and the associated explaining-away mechanisms and sits at the interface between two levels (Fig. 4A).

Known details of the precise connections to thalamus and TRN are consistent with this proposal (*73*). Consider the PGM fragment in Fig. 7D, where the nodes *a*, *b*, and *c* correspond to features at a higher level (V2) and nodes *e*, *f*, and *g* correspond to pools at a lower level (V1). The factor nodes (e.g., node *d*) in this computation implement explaining away, where the feed-forward messages are affected by feedback messages according to equations in Fig. 5E. Explaining away requires feed-forward inhibition (*74*), and our mapping suggests that TRN implements this function as shown in Figs. 6B and 7D. Feedback connections originating from layer 6 project to the inhibitory cells in TRN to modulate the axons of the driving feed-forward connections originating from sensory input or layer 5 of the region below, and passing through TRN. These feedback connections are reciprocal in the sense that they respect the topography of the feed-forward connections (*73*), and they cross connect with feed-forward axons (*75*), matching the requirement for explaining away (*74*). TRN is organized retinotopically enabling it to receive spatially localized feedback information and to influence thalamic relay cells in spatially specific ways (*76*). A detailed view of this circuit between two cortical regions is shown in Fig. 7D. According to Bio-RCN, the explaining-away circuit shown will be a motif that repeats in lateral geniculate nucleus, pulvinar, and, in general, when two cortical areas interact through the thalamus.

Top-down attention gating of various kinds are special cases of explaining-away computation, and the need to support these additional mechanisms provide a rationale for the explaining-away factor being implemented in a separate structure that can act as switchboard for different control signals (*76*). One example of a special case is top-down attention on an object or on a set of features. In the factor graph, this corresponds to setting the parent nodes to be ON, propagating that information everywhere and then letting the feed-forward flow of information to be affected by that. In the neural implementation, this will require sustaining the activity of neurons to be turned on by the top-down attention, which is something that the thalamus could support. Top-down attention to a set of features, or colors, can operate in a similar manner. Lesions in the pulvinar, a higher-order thalamic nucleus, are implicated in deficits in binding object color to object shape using top-down attention (*76*). Other special cases include turning OFF the attention to a particular object or set of features, or reasoning about occlusion and amodal completion. As we pointed out earlier, this kind of top-down object-based or feature-based attention is different from the spatial attention weighting in predictive-coding models.

### Parallel feed-forward pathways

RCN offers rationale for two feed-forward pathways between cortical regions as observed in biology (*73*). The direct cortico-cortical pathway originates in layer 2/3 and projects to layer 4 of the next level, and the indirect cortico-thalo-cortical path originates from layer 5 and is gated through the thalamus and includes explaining away (Fig. 7C). One special case of explaining-away computations is when all parent nodes are assumed to be OFF. In this case, the bottom-up messages are copied to all parent nodes without any feed-forward inhibition. In practically used implementations of RCN, the first forward pass of evidence operates in this manner as well and serves to obtain an approximate but fast inference over higher-level features. For an animal, having a fast, prior-free, feed-forward pathway could be advantageous because it can alert the animal to novel, out-of-context situations. The pathway that goes through the thalamus includes explaining away and attention control and could be slower in switching to a rapid input-driven change. The inhibitory projection from layer 6 to layer 4 might be an approximate version of this explaining-away circuit as well, providing a faster, but approximate, explaining away.

A second justification, consistent with the first, can be offered by the need for keeping imaginations separate from reality. The perceptual apparatus of RCN can be used by a cognitive system (*77*) for visual imagery as part of planning, exploration, and conceptual thinking. A direct feed-forward pathway could offer a mechanism for quickly snapping out of imagination to synchronize back with reality. Mixing imagery with evidence could be behind the hallucinations in schizophrenia, a topic previously explored using probabilistic models (*78*–*81*). The detailed visual circuit offered by RCN could bring further precision to such investigations (*82*), especially those accounts that involve predictive coding (*83*) or active inference (*84*).

### Interaction between shape and appearance (blobs and interblobs)

The factorized contour-surface representation of RCN, which enables generalization to novel combinations of shape and appearance, offers an explanation for the existence of blobs and interblobs (*44*, *85*) in the primary visual cortex and predicts circuit-level details of their interactions. The contour-surface factor in RCN (Fig. 4C) encodes a three way interaction: It enforces continuity of surface properties (e.g., color, texture, surface angle, etc.) between adjacent surface patches except when interrupted by an intervening contour (Fig. 5D). Message passing through this three-way factor enhances the support for contours when the surface patches are discontinuous and vice versa. RCN uses an efficient reparametrization of these message computations that avoids the quadratic set of interconnections required in a naive implementation (see Methods for details). Figure. 6B shows the positioning of the blob interblob interactions within the overall cortical column. Detailed predicted circuit of the interaction between interblob columns and blobs is shown in Fig. 7A, and it uses multiple excitatory neuron types, inhibition, and dendrite-specific gating mechanisms to achieve the required computation.

### Explanations for visual phenomena

RCN was successful in reproducing and explaining three different visual phenomena—subjective contours, neon-color spreading, and occlusion versus deletion effect—to the details of neuron-level activations and their dynamics in different cortical laminae and columns. Notably, all these phenomena are explained as the by-product of inference in a model that was constructed and learned for parsing visual scenes.

#### Subjective contours

**Fig. 8.**
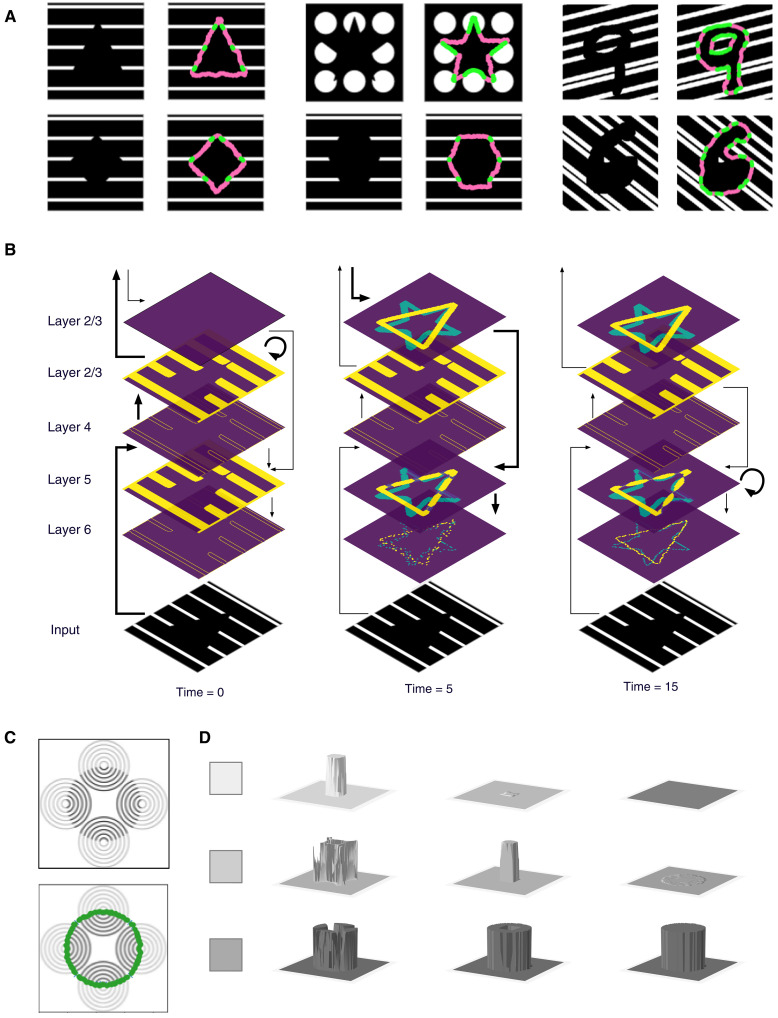
Subjective contours and neon-color spreading. (**A**) Recursive cortical networks (RCN) MAP inference results for stimuli that induce subjective contours in humans. Green, top-down imaginations with bottom-up evidential support. Magenta, contour hallucinations. (**B**) Layer-wise dynamics of subjective contour formation in Bio-RCN. (**C**) Stimulus for neon-color spreading and the subjective contour backtrace. (**D**) Dynamics of color spreading inside the surface, for three different colors.

RCN that is trained to recognize regular shapes “hallucinates” illusory contours in visual stimuli (Fig. 8, A and B) that produce subjective contour perception in people. Subjective contours are an illusion because people see contours that are not present in the input. RCN produce these hallucinations as a natural by-product of “inference to best explanation”—According to the model, evidence in the whole of the image is best explained by hallucinating these contours. In Fig. 8 (A and B), the contours shown in magenta and green constitute the MAP solution at the lowest level of the network, where magenta color indicates contours that exist according to RCN beliefs, although they are not supported by local bottom-up evidence.

The dynamics of subjective contour effects in RCN produce a qualitative match to those observed in biology (Fig. 8C). Physiological results report evidence for neurons in V1 responding to the illusory contour, albeit with a delay compared to the neurons responding to real contours (*6*, *13*, *14*). The temporal dynamics of neuronal responses to subjective contours can be readily understood from the schedule of message propagation. During the forward pass, the features have only local evidence, and hence the neurons in blank spaces do not respond. Once forward pass identifies a potential global percept, that information flows down in the top-down messages to affect the beliefs in lower-level nodes to turn ON some features that were previously OFF.

Bio-RCN’s delineation of feed-forward, lateral, and feedback propagation in shape perception to laminar and columnar precision can help clarify long-standing debates on whether shape perception reduces or enhances activity in the primary visual cortex (*13*, *14*, *86*–*88*). The observation that neural activity is increased in regions with no bottom-up evidence (*88*) is explained by top-down and lateral evidence that is consistent with the global percept, and Bio-RCN’s prediction on this are precise to the level of the specific neuronal populations in laminae and columns. Concurrently, the observation that activity in regions that receive bottom-up input consistent with top-down prediction (*88*) is also explained by Bio-RCN. In the forward pass, all the potential pool and lateral participation of the presence of a feature are active, and feedback and lateral propagation will reduce these activities to those that are consistent with the global percept. Note that this explanation is different from that of “predictive coding” models that require predictions to be subtracted from the input. Bio-RCN also makes lamina-specific predictions that top-down attention will reduce the activity in background elements that were active in the forward pass, which might be useful for designing further experiments on the differentiated roles of different laminae in shape perception (*89*).

#### Neon-color spreading

Inference in RCN reproduced the effect of neon-color spreading, the perception of an illusory surface with an illusory color (*90*), and its cortical implementation predicts circuit dynamics at the neuronal level (Fig. 8D). The suggested mechanism behind these effects is the interplay between boundary completion and surface filling-in in visual cortex (*15*). Notably, the filling in of the illusory surface respects the boundaries of the illusory contours. The neon-color spreading effect is a natural by-product of the dynamics of MAP inference in RCN. Three-way interactions in the contour-surface MRF encourage continuity between adjacent surface patches unless the intervening contour node is turned ON. A forward pass through this model produces approximate edge and surface responses and leads to the selection of “circle” as the best top-level hypothesis. The backward pass, which is based on selecting the most active hypothesis at the top level of the contour hierarchy, will then enforce the corresponding contour discontinuities on the surface CRF. Similar to the subjective contour effects described earlier, the stimulus shown in Fig. 8D has sufficient local edge evidence to support a circle as the top level hypothesis in the RCN contour hierarchy and to hallucinate the missing contours of the circle. The top-down partial MAP configuration for contours of the circle, including the hallucinated portions, then influences the propagation in the CRF. The discontinuity imposed by the top-down contours will propagate in the CRF with further message passing to create the fill-in effect with the dynamics shown in Fig. 8D.

The contour formation (Fig. 8B) and the surface filling dynamics in (Fig. 8D) Bio-RCN match the dynamics and laminar profiles of figure-ground segregation observed in cortical layers (*91*). Matching biological observations, layer 4 neurons are activated first, followed by layer 2/3 in the feed-forward pass. Labeling of the perceived figure with enhanced activity occurs in layers 2/3 and layer 5, and region-filling is determined by the layers that receive feedback information from higher level (*89*, *91*, *92*). Together, neon-color spreading in Bio-RCN supports the observation that object completions involve feedback loops that integrate contour interpolation with surface filling in (*93*).

#### Occlusion versus deletion

**Fig. 9.**
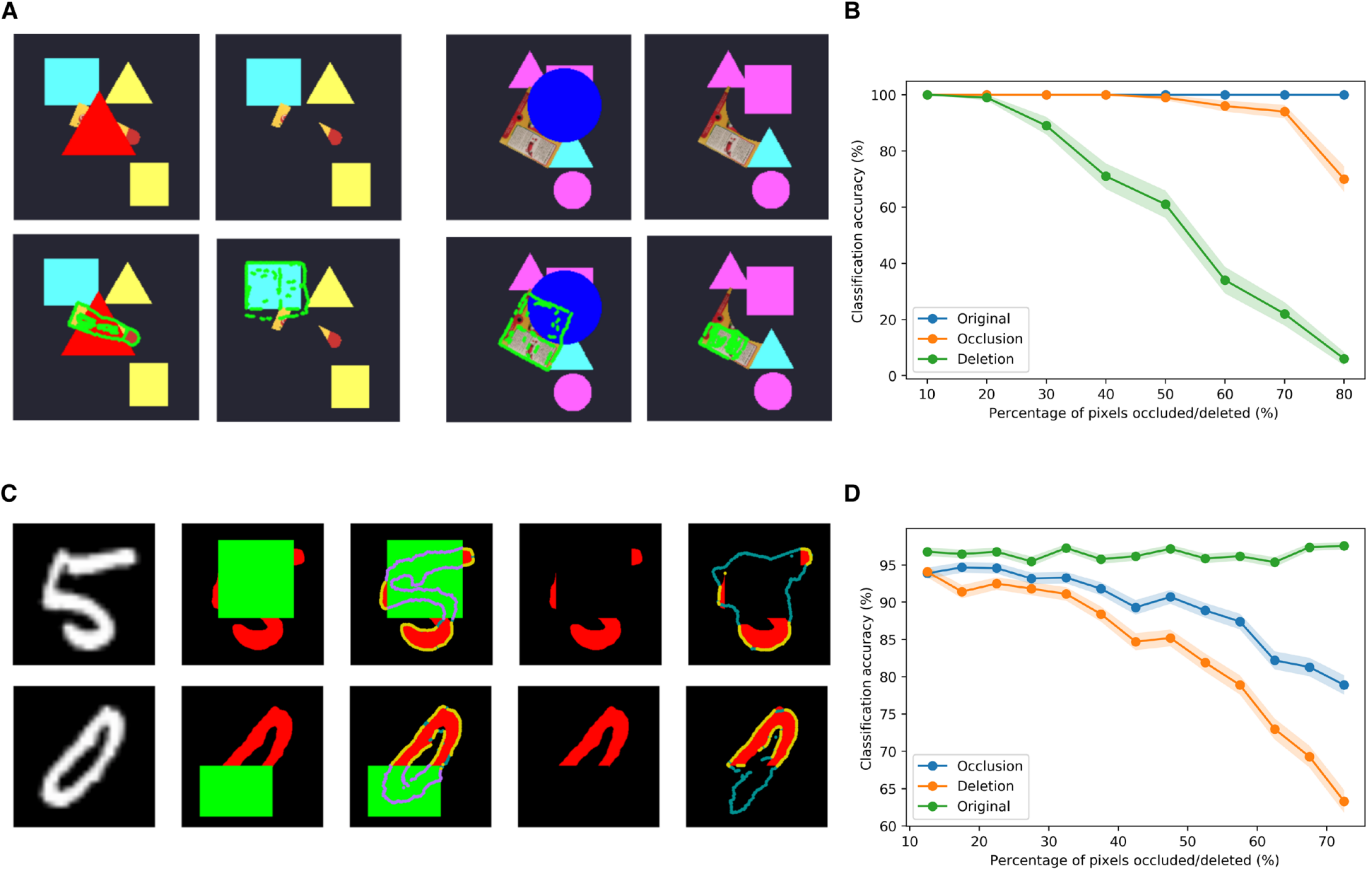
Occlusion versus deletion. (**A**) Humans have a harder time perceiving objects with deleted portions compared to the case where the same portions are occluded. Clockwise in each set of four images: (i) Object with occlusion, (ii) the same object with occluded portion deleted, (iii) recursive cortical networks (RCN) MAP inference for occlusion case, (iv), RCN MAP inference for deletion. (**B**) Recognition accuracy for occlusion versus deletion as a function of the amount of missing evidence. (**C**) Occlusion versus deletion for MNIST digits. In each row left to right, original digit, occluded digit, MAP inference overlaid on occlusion, digit with occluded portions deleted, MAP inference with deletion. (**D**) The same as (B) for MNIST digits.

RCN also reproduces the psychophysics observation (*94*) that humans are much better at detecting objects under occlusion than the same objects where the same regions have been deleted instead of occluded, while keeping identical visible portions (Fig. 9). Occlusion reasoning performance of RCN was already demonstrated in (*10*), and the RCN generative model offers the reasoning for why partially occluded images are easier to recognize compared to their partially deleted counterparts. Deletion of the parts of an object is absence of evidence for those parts. When those same parts are missing because of occlusion, the model can explain away the absence of evidence as occlusion. Mechanistically, the portions that are deleted will contribute negative evidence to the overall hypothesis if there is no occlusion to explain their absence. If the missing parts of an objects are filled by other objects, then the explaining-away computations will result in the missing evidence being treated as “uncertain evidence” (log-likelihood = 0) as opposed to negative evidence. The dynamics of occlusion reasoning and filling in have limited studies in neuroscience (*95*–*97*), and predictions from Bio-RCN might be a useful tool in further explorations.

## DISCUSSION

Here, we derived a detailed and functional mathematical theory of cortical and thalamic microcircuits underlying visual perception. The approach we followed can be construed as a general methodology by which we can seek to understand cortical function. The first step in this methodology is to triangulate between cortical data, properties of the world, and computational principles to build algorithmic models that are tested on real-world tasks. The second step considers the algorithmic model in conjunction with anatomical and physiological data to derive detailed and functional microcircuit-level models. The advantage of this approach is that the derived circuit is methodology constrained to a coherent functional model that cuts across different organizational levels—cortical regions, thalamus, cortical columns, cortical laminae, neuron types, and dendrites.

Our approach is in contrast to large-scale systems neuroscience efforts gathering large amounts of data on cortical connectivity (*5*, *98*, *99*). While more data are definitely useful, purely data-driven simulations are an unlikely path to understanding cortical function. We believe that the neuroscience-guided theoretical approach we use here is a necessary complement to data-gathering and experimental approaches.

Message-passing inference in RCN, the algorithmic base for the microcircuit model, is more general and more effective than typical predictive coding (*11*) accounts. Complex problems like perception might require more general nonparameteric inference compared to predictive coding that makes restrictive linear/Gaussian assumptions. Our experiments demonstrate that various accounts of response reduction and enhancement can be accounted using our more general generative model without the restrictive assumptions of predictive coding (*28*).

While known neuroanatomy and physiology impose constraints on cortical implementation of a set of inference equations, several variations of the same implementation are possible because neurons can be moved to different laminae or a computation can be accomplished in dendrites instead of neurons. Future research could explore this hodology in more detail and combine it with wiring and metabolic constraints. Variations in implementation of one circuit will also affect the implementation of upstream and downstream circuits due to mutual constraints. The biological implementation we proposed here should be taken as a starting point, not the final result, for such investigations. Further neuroscience experiments could test the veracity of these predictions and suggest alternative implementations. Predictions from our circuit model could also help further investigations of neurological and psychiatric conditions (*78*, *80*).

RCN currently is a model of the ventral visual pathway. While many aspects of the derived microcircuit are likely to generalize to other visual pathways, some aspects like blob-interblob interactions are likely to be specific to the ventral pathway. RCN could potentially be extended to use temporal information for pooling (*24*) and to use binocular disparity for depth perception. In future work, we intend to explore those extensions and study how they might result in a more comprehensive model of visual cortical microcircuits.

We started with an algorithmic AI model that was inspired by observations from neuroscience and showed how that model can be used for deriving neuroscience insights. Such interactions between artificial intelligence and neuroscience research are likely to simultaneously help accelerate progress in both fields toward two ultimate goals: building artificial general intelligence and understanding the brain.

## METHODS

### BP updates with temperature

A set of random variables connected through factors form a factor graph and thus a graphical model. The values of the factors define a joint probability distribution over the random variables. This probability distribution might be unnormalized, in which case, a normalization constant must be applied to obtain a proper distribution.

In more detail, a PGM parameterized by θ can be written in terms of its factors as $\text{log}p\left(x\right)=\sum_{a}f_{a}\left(x_{a}\right)-\text{log}Z\left(\theta \right)$, where the factors are indexed by *a*, and $x_{a}$ is the collection of random variables connected to factor $f_{a}$. The necessary normalization constant logZ(θ) is called the log-partition function and can be computed as $\text{log}Z\left(\theta \right)=\text{log}\sum_{x}\text{exp}\left[f_{a}\left(x_{a}\right)\right]$.

In the context of this work, inference refers to computing either the marginals of all variables or the maximum probability configuration. Both types of inference can actually be subsumed in the same framework by using a temperature variable. Using variational arguments, we know that the log-partition function can be lower-bounded as

$$\text{log}Z\left(\theta \right)\geq \sum_{a}E_{q\left(x_{a}\right)}\left[f_{a}\left(x_{a}\right)\right]+TH\left[q\left(x\right)\right]$$

where *q*(*x*) is an arbitrary probability density function (PDF) and $q\left(x_{a}\right)$ is the marginal of that PDF over the subset of variables connected to factor *a*, $x_{a}$. H[q(x)] is the entropy of the PDF. In its standard presentation, the variational inequality is shown with a fixed temperature *T* = 1. When a temperature 0 < *T* ≤ 1 is used, the inequality still holds (note that for discrete variables, the entropy is always positive), but its properties change.

In particular, consider what happens if we try to maximize the right hand side (r.h.s.) with respect to (w.r.t.) *q*(*x*) for *T* = 1. It is a standard result that the maximizing *q*(*x*) is actually *p*(*x*) and that for that choice, the inequality becomes tight, i.e., both sides of the inequality become identical. However, when we set *T* → 0, the regularization of the entropy term vanishes and we are left with an expectation over an unnormalized logp(x). Obviously, maximizing w.r.t. *q* (*x*) in this situation will lead to a Dirac delta placed at the maximum of the logp(x). In other words, for *T* = 1, the maximization leads to $q\left(x_{a}\right)$ containing the marginals of *p*(*x*) (which can be further marginalized to obtain the marginals of individual variables), whereas for *T* → 0, we recover the globally maximizing configuration of *p*(*x*) in the form of a Dirac delta at the maximizing configuration. This turns marginalization (which requires summing exponentially many terms) and the discovery of the global maximizing configuration (which requires evaluating exponentially many configurations) into the same continuous maximization problem, with a temperature parameter selecting the desired behavior.

Obviously, the exact maximization w.r.t. *q*(*x*) is not less challenging than the original problems. However, we can make progress by introducing two approximations. The first one is to replace the entropy of the entire distribution with a modular approximation called the Bethe entropy

$$H_{B}\left[q\left(x\right)\right]=\sum_{a}H\left[q,\left(x_{a}\right)\right]+\left(1-n_{i}\right)\sum_{i}H\left[q,\left(x_{i}\right)\right]$$

where $x_{i}$ are each of the variables of the PGM and $n_{i}$ is the number of factors that each variable is connected to. This approximation is exact for trees and often close to the correct one in practice. In general, it is neither an upper nor a lower bound on the true entropy. The second approximation is to consider all the $q\left(x_{a}\right)$ as independent distributions over subsets of variables that have consistent marginals $q\left(x_{i}\right)$ on each variable. This condition is not enough to guarantee that a global distribution *q*(*x*) with those marginals will exist, so we call these $q\left(x_{a}\right)$ pseudomarginals. Again, in the case of a tree PGM, this condition is enough to guarantee that a consistent global *q*(*x*) does exist.

With those two approximations, and reversing the sign, we obtain a much more tractable problem, the constrained minimization of the Bethe free energy where we have included the temperature parameter

$$\begin{array}{c} \underset{q\left(x_{a}\right),q\left(x_{i}\right)}{\text{min}}-\sum_{a}E_{q\left(x_{a}\right)}\left[f_{a}\left(x_{a}\right)\right]\\ -T\left\{\sum_{a}H\left[q\left(x_{a}\right)\right]+\left(1-n_{i}\right)\sum_{i}H\left[q\left(x_{i}\right)\right]\right\} \end{array}$$

$$s.t.\sum_{x_{a\backslash i}}q\left(x_{a}\right)=q\left(x_{i}\right)\sum_{x_{i}}q\left(x_{i}\right)=1\;q\left(x_{i}\right)\geq 0$$

The proper Bethe free energy corresponds to the choice *T* = 1. When working with the standard variational formulation above (using the Shannon entropy), we are almost always interested in the *T* = 1 setting. This is a convenient setting for learning (avoids overfitting), makes the lower bound tighter, and keeps the desirable property of being an “exclusive” Kullback-Leibler (KL) divergence (roughly meaning that the approximate posterior will avoid placing high probability in regions that are low probability according to the true posterior). However, when working with the Bethe free energy, the above considerations are not necessarily true, and introducing a temperature parameter can give us more flexibility. First, the Bethe entropy does not necessarily lower bound the Shannon entropy. This means that the Bethe free energy no longer lower bound the partition function. This, together with the use of pseudomarginals (which no longer necessarily correspond to a valid joint posterior distribution) results, for the *T* = 1 setting in an approximate “inclusive KL” divergence (*100*). This means that the approximate posterior for *T* = 1 can place mass in a region in which the true posterior has very low or zero probability. While this might be desirable to avoid overfitting in a learning setting, it might be undesirable when running inference. In cases in which this is to be avoided, we can reduce the value of *T*. As *T* → 0, the above Bethe free energy becomes again a lower bound on the marginal likelihood, and the behavior of the approximate posterior becomes again exclusive. As we will see below, we also recover a different, well-known version of BP.

We can turn this constrained optimization problem into its dual form by forming the Lagrangian. If we differentiate w.r.t. the dual parameters and set them to zero, we obtain a set of self-consistent updates known as BP. These dual parameters behave as messages, propagating information between factors and variables. We have two types of messages—those that go from factors to variables and those that go from variables to factors. Iterating these updates, we approximately minimize the Bethe free energy. Recall that the two approximations introduced in the Bethe free energy are actually exact for the case of tree PGMs; it turns out that in this case, the Bethe free energy is convex and this update scheme results in exact minimization. The updates are

$$m_{i\to a}\left(x_{i}\right)\overset{c}{=}\sum_{b\in N\left(i\right)\backslash a}m_{b\to i}\left(x_{i}\right)$$ (1)

$$m_{a\to i}\left(x_{i}\right)\overset{c}{=}T\text{log}\sum_{x_{j\in a\backslash i}}f_{a}{\left(x_{a}\right)}^{\frac{1}{T}}\text{exp}\left[\frac{\sum_{j}m_{j\to a}\left(x_{j}\right)}{T}\right]$$ (2)

$$b(x_{i})\overset{c}{=}\sum_{b\in N\left(i\right)}m_{b\to i}\left(x_{i}\right)$$ (3)

**Fig. 10.**
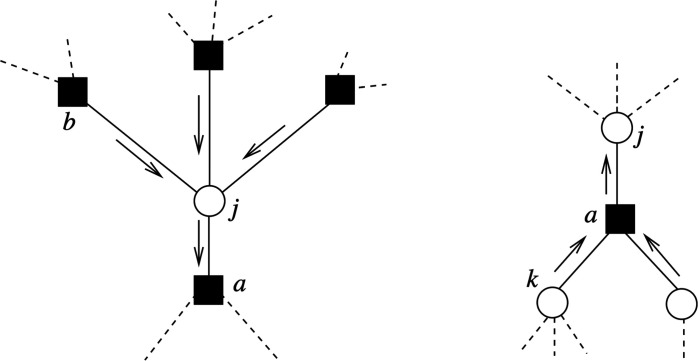
Illustration of the two types of message updates. On the left, update (Eq. 1) (messages from variables to factors), independent of the temperature or the factor definition; on the right, update (Eq. 2) (messages from factors to variables), with the form of the update being specific to each factor and temperature.

The update (Eq. 1) updates the messages from the variables to the factors (see the left of Fig. 10); the update (Eq. 2) updates the messages from the factors to the variables (see the right of Fig. 10). Last, Eq. 3 provides the beliefs for each variable. At convergence, the beliefs, once properly normalized, are an approximation to the marginal $q\;\left(x_{i}\right)$ (for *T* = 1) or to the max-marginal (for *T* → 0), i.e., to obtain an approximately maximizing configuration, we iterate these updates to convergence with *T* → 0 and choose for each variable the assignment $x_{i}$ that maximizes $b\;\left(x_{i}\right)$.

These updates are intended to be performed sequentially but also produce good empirical results when run in parallel if some amount of damping is used. Although more sophisticated approaches to minimize the Bethe free energy exist, this is one of the fastest, with very little degradation in performance in practice.

These updates can be used for any value 0 < *T* ≤ 1 and take the name sum-product (or simply BP) for *T* = 1 and max-product (also max-sum or belief revision) for *T* → 0. Also notice that for the latter case, update (2) can be simply expressed as

$$m_{a\to i}\left(x_{i}\right)\overset{c}{=}\underset{x_{j\in a\backslash i}}{\text{max}}f_{a}\left(x_{a}\right)+\sum_{j}m_{j\to a}\left(x_{j}\right)$$ (4)

Last, here are some practical considerations about the storage of messages in practice. First, observe that each message is actually a function of the values that variable $x_{i}$ (which they originate from or which they go to) can take. Therefore, in our case of discrete variables, each message is actually a vector. Second, note that those vectors are defined in the update equations up to a normalization constant. Although any normalization constant is mathematically valid and would not change the algorithm, in practice, it is beneficial to normalize to avoid precision loss. Two typical normalization choices would be to subtract the maximum of the vector or to subtract the value that would make the message represent a log-probability density, i.e., achieving $\sum_{x_{i}}\text{exp}\left[m_{a\to i}\left(x_{i}\right)\right]=1$. In the case of binary variables, a good option is to simply store them as a single real number by subtracting $m_{a\to i}\left(x_{i}=0\right)$ from both elements of the vector. In this way, we know that the value of the message for *x_i* = 0 is always 0, and we only need to store a single real value

$$m_{a\to i}=m_{a\to i}\left(x=1\right)-m_{a\to i}\left(x=0\right)$$

### Factor definitions

Here, we provide the precise definitions for the factors used in this work and the corresponding message updates (resulting from applying Eq. 2 to the definition) plus any other relevant comments. The messages involving binary variables will be defined as single real values, following the normalization of the previous section.

### Edge-appearance factor

The edge-appearance factor connects two categorical variables, *a* and *b*, describing the appearances on both sides of an edge. The presence of the edge itself will be a binary variable *e*. The factor is defined as follows

$$f\left(a,b,e\right)=\left\{\begin{array}{cc} 0 & \text{if}\;a=b\;\text{and}\;e=0\\ -c & \text{if}\;a\neq b\;\text{and}\;e=1\\ -\infty & \text{otherwise} \end{array}\right.$$

so that the lack of an edge forces appearance continuity and its presence forces appearance discontinuity (with a penalty c>0 added). If we naively plug this factor into Eq. 2 to obtain, e.g., the message from the factor toward *b*, we would obtain

$$m_{f\to b=i}\overset{c}{=}T\text{log}\sum_{j,k}f{\left(a=j,b=i,e=k\right)}^{\frac{1}{T}}\text{exp}\left(\frac{m_{e=k\to f}+m_{a=j\to f}}{T}\right)$$

which, while correct, takes $O\left(N^{2}\right)$ computation for *N* categories in *a* and *b* if naively implemented. A more efficient way to write this update is to first compute some intermediate values $s_{a},s_{b},s_{d}$ and then reuse them. The following equations provide all of the factor to variable updates (including the one above, with the same numerical value) but written in a way in which their computation takes only O(N) time to compute

$$s_{a}=T\text{log}\sum_{i}\text{exp}\left(\frac{m_{a=i\to f}}{T}\right)$$

$$s_{b}=T\text{log}\sum_{i}\text{exp}\left(\frac{m_{b=i\to f}}{T}\right)$$

$$s_{d}=T\text{log}\sum_{i}\text{exp}\left(\frac{m_{a=i\to f}+m_{b=i\to f}}{T}\right)$$

$$\begin{array}{c} m_{f\to a=i\overset{c}{=}}m_{b=i\to f}\\ +T\text{log}\left\{1+\text{exp}\left(\frac{m_{e\to f}}{T}-c\right)\left[\text{exp}\left(\frac{s_{b}-m_{b=i\to f}}{T}\right)-1\right]\right\} \end{array}$$

$$\begin{array}{c} m_{f\to b=i}m_{a=i\to f}\\ +T\text{log}\left\{1+\text{exp}\left(\frac{m_{e\to f}}{T}-c\right)\left[\text{exp}\left(\frac{s_{a}-m_{a=i\to f}}{T}\right)-1\right]\right\} \end{array}$$

$$m_{f\to e=1}=T\text{log}\left[\text{exp}\left(\frac{s_{a}+s_{b}-s_{d}}{T}\right)-1\right]-c\;\left(m_{f\to e=0}=0\right)$$

### OR factor

The OR factor connects multiple binary inputs, $\left\{c_{1},c_{2},\;\ldots \;,c_{M}\right\}$, to the result of ORing them, *e*, which is also binary. The precise mathematical definition is

$$f\left(c_{1},c_{2},\;\ldots \;,c_{M},e\right)=\left\{ \begin{array}{cc} 0 & \text{if}\;\text{OR}\left(c_{1},c_{2},\ldots ,c_{M}\right)=e\\ -\infty & \text{otherwise} \end{array}\right.$$

i.e., this factor ensures that the output variable *e* is indeed the OR of the input variables $\left\{c_{1},c_{2},\ldots ,c_{M}\right\}$.

As in the previous case, we can plug this factor definition into Eq. 2 to obtain the message updates. Again, we can save some computation by defining the intermediate variables $z_{m}$ and $h_{m}^{-}$ and reusing them. Thus, the updates are

$$z_{m}=-T\text{log}\sigma \left(-\frac{m_{c_{m}\to f}}{T}\right)$$

$$h_{m}^{-}=\sum_{j\neq m}z_{j}+\mathrm{Tg}\left(\sum_{j\neq m}\frac{z_{j}}{T}\right)$$

$$m_{f\to e}=\sum_{m}z_{m}+\mathrm{Tg}\left(\sum_{m}\frac{z_{m}}{T}\right)$$

$$m_{f\to c_{m}}=-T\text{log}\left[\sigma \left(\frac{h_{m}^{-}}{T}\right)+\sigma \left(-\frac{h_{m}^{-}}{T}\right)\text{exp}\left(-\frac{m_{e\to f}}{T}\right)\right]$$

with g(x)=log[1−exp(−x)]

### POOL factor

The POOL factor connects a binary “pooling” variable, *a*, to *M* binary mutually exclusive options, $\left\{b_{1},b_{2},\ldots ,b_{M}\right\}$, that can activate only when the pooling variable is on, each with uniform probability 1/*M*. The formal definition of this factor is

$$\begin{array}{c} f\left(a,b_{1},b_{2},\ldots ,b_{M}\right)\\ =\left\{ \begin{array}{cc} 0 & \text{if}\;a=0\;\text{and}\left\{b_{m}=0\right\}\forall_{m}\\ -\text{log}\left(M\right) & \text{if}\;a=1\;\text{and exactly}\;\text{one}\;\text{of}\;\left\{b_{1},b_{2},\ldots ,b_{M}\right\}\;\text{is equal to}\;1\\ -\infty & \text{otherwise} \end{array}\right. \end{array}$$

Since in this case, we are only interested in the max-product setting in which *T* → 0, we can plug in the POOL factor definition in Eq. 4 to obtain the message updates directly as

$$m_{f\to a}=\underset{m\in \left\{1,\ldots ,M\right\}}{\text{max}}m_{b_{m}\to f}-\text{log}\left(M\right)$$

$$m_{f\to b_{m}}=\text{min}\left[m_{a\to f}-\text{log}\left(M\right),-\underset{j\neq m}{\text{max}}m_{b_{j}\to f}\right]$$
